# Supplementary material for: Maternal Immunization with Adjuvanted Recombinant Receptor-Binding Domain Protein Provides Immune Protection against SARS-CoV-2 in Infant Monkeys
Source: Vaccines (Basel). 2024 Aug 20;12(8):929. doi: 10.3390/vaccines12080929 (PMC11359192; doi:10.3390/vaccines12080929)
Supplement: Supplementary file 1 [file vaccines-12-00929-s001.zip › vaccines-3114130-supplementary.pdf]

Supplementary Materials

Coe, C.L.; Nimityongskul, F.; Lubach, G.R.; Luke, K.; Rancour, D.; Schomburg, F.M. Maternal immunization with adjuvanted recombinant receptor-binding domain protein provides immune protection against SARS-CoV-2 in infant monkeys. *Vaccines* **2024**

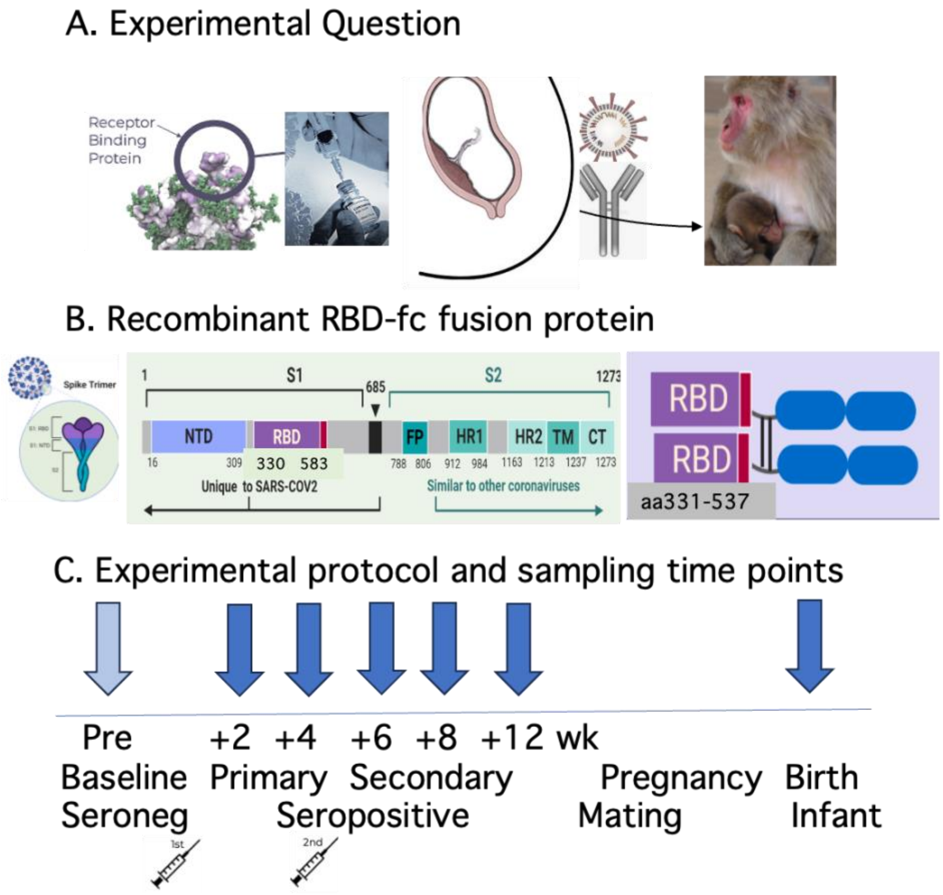

Figure S1. Illustration of the primary research question, the recombinant fusion protein used for immunization, and time points for collecting blood samples. **A.** The main aim was to verify that immunization of female monkeys with adjuvanted recombinant RBD-fc preconception or during early pregnancy would result in the transfer of maternal RBD-specific IgG to their infants. **B**. SARS-CoV-2 structure and visual representation of amino acids (aa331-537) in the RBD-fc fusion protein. **C.** Blood collection schedule showing the samples used to track the antibody response to the two immunizations administered preconception to 5 adult female monkeys. The pregnant female was immunized only once, but the serial collection of blood from her was the same. After birth, blood samples from all 12 mothers and infants were collected similarly and used to quantify the transfer of maternal RBD-specific IgG to the infant. The baseline sample collected prior to vaccination verified that all adult monkeys were seronegative before the RBD-fc was administered; none had antibody to S1 subunit, S2 subunit and nucleotide antigens. These samples also provided Negative control sera, in addition to the other Negative and Positive controls, included in the CSA and ACE2 binding inhibition assays.

Table S1. Antibody levels for the SARS-CoV-2 S1 and S2 subunits, as well as nucleocapsid antigens, in 6 previously immunized female rhesus monkeys and their infants after birth. IgG levels were quantified with the Colony Surveillance Assay SARS-CoV-2 kit, and titers are expressed in relative intensity units (RIU). Immunization of the mothers with recombinant RBD-Fc elicited antibody responses that were specific to the viral binding domain in the S1 subunit. This immunogenic specificity was also evident in the maternal IgG was transferred placentally to the infants prior to delivery.

	S1 subunit	S2 subunit	Nucleocapsid
Mothers	**19,883 (2560)***	48 (28)	258 (124)
Infants	**19,188 (2752)***	87 (31)	157 (31)
Negative Control**	0	1617	276
Positive Control**	42,507	46,304	44,407

*IgG levels higher than the cutoff for seropositivity were found only for the S1 subunit in the previously immunized females and their infants. These values are highlighted with Bold font.

**Experimental samples were run at 3 dilutions and the mean (SE) titers are shown for the 1:10,000 dilution. Negative and positive assay controls were run at a 1:200 dilution and were used to confirm the accuracy of the assay's performance across runs. The 12 maternal and infant samples were assayed on the same plate. The Assay Control values are from the seronegative and seropositive sera that were included on this plate.

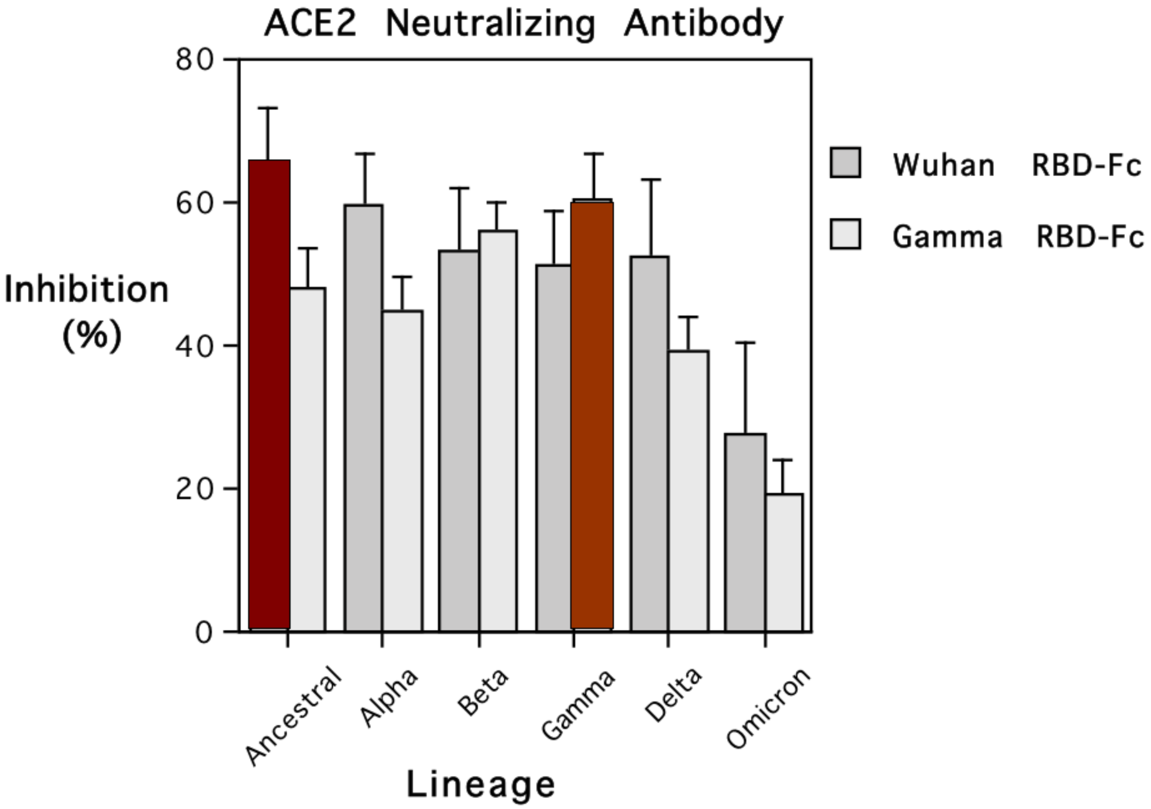

Figure S2. Neutralization of SARS-CoV2 and RBD antigen binding to angiotension-converting enzyme 2 (ACE2). A multiplex panel including 6 variants was used (Meso Scale Discovery, MSD, Rockville Maryland). The amount of inhibition reflected the type of RBD-Fc that had been administered to the adult females. Both maternal and infant sera showed a similar pattern of immunogenic specificity. More inhibition of ACE2 binding of Wuhan RBD was evident for sera from infants born to the adult females administered Wuhan RBD-Fc. Conversely, more inhibition of antigen binding to the Gamma variant was evinced by sera from infants born to mothers who had been previously administered Gamma RBD-Fc antigens. The variant-specific differential is shown by red coloring to highlight the concordance between RBD-Fc used for immunization and the viral variant tested in the *in vitro* assay. Both maternal and infant sera showed significantly less neutralization when tested against the later viral variants, Delta and Omicron. As a Negative control, sera from non-immunized, seronegative monkeys (n=12) were included in the assay and elicited negligible binding inhibition (<1%). Two Positive controls were also included in the assay. The sera from the immunized mothers and their infants showed BI above the levels exhibited by both anti-SARS-CoV-2 spike RBD neutralizing antibody and a pooled human SARS CoV-2 national IgG positive standard at the dilutions tested.
